# mTOR signaling controls protein aggregation during heat stress and cellular aging in a translation- and Hsf1-independent manner

**DOI:** 10.1016/j.jbc.2025.108172

**Published:** 2025-01-10

**Authors:** Arthur Fischbach, Per O. Widlund, Xinxin Hao, Thomas Nyström

**Affiliations:** Institute for Biomedicine, Sahlgrenska Academy, Centre for Ageing and Health-AgeCap, University of Gothenburg, Gothenburg, Sweden

**Keywords:** aging, proteostasis, protein aggregation, mTOR, GATOR, spatial protein quality control, proteinopathy

## Abstract

The mechanistic target of rapamycin (mTOR) signaling pathway appears central to the aging process as genetic or pharmacological inhibition of mTOR extends lifespan in most eukaryotes tested. While the regulation of protein synthesis by mTOR has been studied in great detail, its impact on protein misfolding and aggregation during stress and aging is less explored. In this study, we identified the mTOR signaling pathway and the linked Seh1-associated complex as central nodes of protein aggregation during heat stress and cellular aging, using *Saccharomyces cerevisiae* as a model organism. Based on a synthetic genetic array screen, we found that reduced mTOR activity, achieved through deletion of *TCO89*, an mTORC1 subunit, almost completely prevents protein aggregation during heat stress and aging without reducing global translation rates and independently of an Hsf1-dependent stress response. Conversely, increased mTOR activity, achieved through deletion of *NPR3*, *a* Seh1-associated complex subunit, exacerbates protein aggregation, but not by overactivating translation. In summary, our work demonstrates that mTOR signaling is a central contributor to age-associated and heat shock-induced protein aggregation, and that this is unlinked to quantitatively discernable effects on translation and Hsf1.

Aging is hypothesized to be a result of accumulated cellular damage over time (1, 2, 3). In the budding yeast *Saccharomyces cerevisiae*, aging is associated with the accumulated cellular damage in the form of protein aggregates, oxidatively damaged proteins, defective mitochondria and vacuoles, as well as extrachromosomal ribosomal DNA circles (4, 5). The mechanistic target of rapamycin (mTOR) signaling pathway is central to the aging process (6). Genetic or pharmacological inhibition of mTOR extends lifespan in most eukaryotes tested, including *S. cerevisiae*, *Caenorhabditis elegans*, *Drosophila melanogaster*, or *Mus musculus* (6). In *S. cerevisiae*, perturbations in mTOR affects both replicative (7, 8) and chronological (9) aging. Replicative aging in yeast is defined by the maximum number of daughter cells a mother cell can produce before becoming senescent, while chronological aging measures the time a nondividing yeast cell can survive. mTOR functions are mainly attributed to the mTORC1 complex, which contains either Tor1 or Tor2, together with Tco89, Kog1, and Lst8 (10). The Seh1-associated (SEA) complex, consisting of the SEACAT and SEACIT subcomplexes, regulates mTOR activity. SEACIT, composed of Npr2, Npr3, and Sea1, inhibits mTOR, while SEACAT, containing Sea2-4, Sec13, and Seh1, stimulates the mTOR activity (11). Recent structural determination of the SEA complex revealed that SEACAT acts as a scaffold for the binding of mTOR regulators rather than directly inhibiting SEACIT (12). The mammalian ortholog of the SEA complex, known as GATOR, consists of GATOR1 and GATOR2 subcomplexes with inhibitory and stimulating effects on mTOR activity, respectively (13). mTOR primarily controls anabolic processes such as lipid, nucleotide, and protein synthesis.

When protein quality control (PQC) fails, misfolded proteins and protein aggregates form. These aggregates are detrimental to the cell due to the loss of function of the aggregating proteins or gain of toxic, noncanonical functions of the formed aggregates. PQC failure can lead to neurodegenerative diseases in humans, where aggregated proteins impair cellular functions and eventually cause cell death (14). In some organisms, protein aggregates can be cleared by “disaggregases” of the Hsp100 family, such as Hsp104 in yeast (15, 16, 17, 18, 19, 20). When such clearance fails, aggregates are sequestered into large inclusions at specific cellular sites by a process referred to as spatial PQC. This sequestration may reduce the toxicity of such aggregates, by decreasing the exposed surface area, restricting interactions with other functional proteins, and limiting titration of PQC components, including chaperones (21, 22). In addition, it has been demonstrated that inclusions associated with the mitochondria are cleared out faster than inclusions that are not (23), indicating that specific locations in the cell may be more efficient in certain PQC processes.

In yeast, misfolded and aggregated proteins initially accumulate at multiple sites, known as stress foci, CytoQs, or Q-bodies (24, 25, 26) throughout the cytosol and at the surface of various organelles, such as the mitochondria, vacuole, and the endoplasmic reticulum. Upon prolonged proteostatic stress, these aggregates coalesce into larger inclusions by an energy-dependent process (24). These inclusions are categorized by their proximity to specific organelles (peripheral vacuole-associated insoluble protein deposit proximal to the vacuole (24, 27); juxtanuclear quality control site on the cytosolic side of the nuclear membrane (27); intranuclear quality control site in close proximity to the juxtanuclear quality control site, but within the nucleus, next to the nucleolus (26); and a site adjacent to the mitochondria (28)). Different sorting mechanisms and factors appear to be involved in sorting misfolded proteins to each specific site (26, 29, 30). Most misfolded proteins studied are sequestered to all these sites (24, 27, 31). However, the vacuole-associated insoluble protein deposit (25) seems to be the deposition site for amyloid proteins. Mammalian cells sequester aggregated proteins in a deposition site near the centrosome, known as the aggresome (32, 33). The importance of spatial control of aggregates for cellular fitness, rejuvenation, and longevity is primarily inferred from results using mutations that cause defects in spatial PQC (18, 19, 34, 35) but the physiological significance of the spatial deposition of aggregates is far from clear.

While the regulation of protein synthesis by mTOR is well understood, its impact on protein misfolding and aggregation is less clear. In this study, we identified the mTOR signaling pathway as a central node controlling protein aggregation during heat stress and cellular aging. Using a genome-wide genetic screen, we demonstrated that reduced mTOR activity prevents protein aggregation during heat stress and aging without reducing global translation rate and independently of an Hsf1-dependent stress response. Conversely, increased mTOR activity exacerbates protein aggregation in the absence of increased translation. Our data demonstrate that mTOR signaling and, in particular, the SEA complex are central contributors to the control of protein aggregation during heat stress and cellular aging.

## Results and discussion

### Screening for modulators of protein aggregation

**Figure 1:**
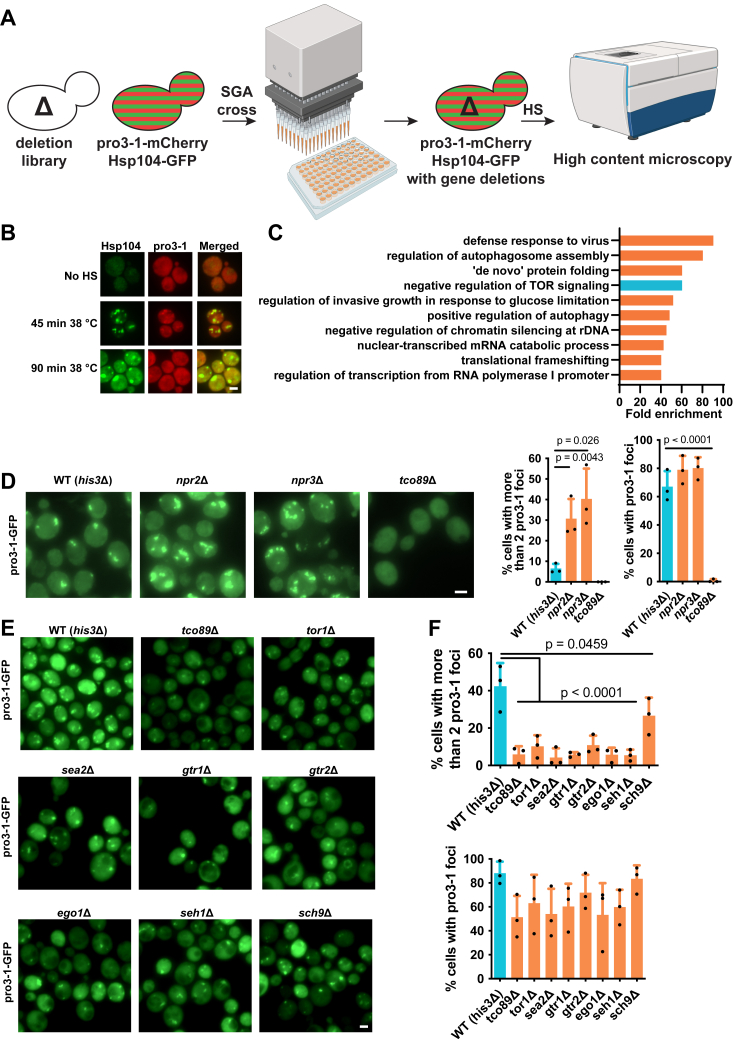
**Screen for identifying modulators of pro3-1 protein aggregation.***A*, screen strategy. Yeast cells expressing pro3-1-mCherry and Hsp104-GFP were crossed with the yeast deletion library using synthetic genetic array (SGA) technology. The resulting strains were subjected to a 38 °C heat shock (HS) for 45 or 90 min followed by fixation. Fluorescence images were generated with high content microscopy. Created with BioRender.com. *B*, example images from the screen. *C*, GO analysis of significant biological process enrichments with the hits from the screen for restricting pro3-1 aggregation. *D*, representative fluorescence microscopy images of manually generated yeast strains with gene deletions in the mTOR signaling regulators *NPR2*, *NPR3*, or *TCO89* expressing pro3-1-GFP. Cells were subjected for 90 min to a 38 °C HS. Quantifications of cells with pro3-1 aggregates are shown on the *right* side. Data are presented as mean values ± SD. *n* = 3 independent experiments. One-way ANOVA followed by Dunnett’s multiple comparison test. *E*, representative fluorescence microscopy images of deletion strains of more positive mTOR regulators expressing pro3-1-GFP. Cells were subjected for 45 min to a 38 °C HS. *F*, quantification of the percentage of cells with pro3-1 aggregates from *E*. Data are presented as mean values ± SD. *n* = 3 experiments. One-way ANOVA followed by Dunnett’s multiple comparison test. Scale bars represent 2 μm. mTOR, mechanistic target of rapamycin; GO, Gene Ontology; HS, heat shock.

To discover modulators of protein aggregation during a heat shock, we conducted a comprehensive genome-wide screen. To this end, we used the yeast deletion library together with the model misfolding reporter pro3-1-mCherry (36) and synthetic genetic array (SGA) technology (Fig. 1*A*). To validate that the foci pro3-1 forms upon heat stress are *bona fide* protein aggregates, we expressed the protein aggregate marker Hsp104-GFP in the same cells (Fig. 1*B*). Our initial analysis focused on identifying genes whose deletion resulted in increased protein aggregation postheat shock (Supplementary data 1). During heat shock, protein aggregates form and gradually coalesce into one or two foci over time. We selected a 90-min heat shock duration to allow sufficient time for aggregate coalescence, thereby making any defects more discernible. Some of the functional Gene Ontology (GO) groups identified as important in restricting aggregates were anticipated, including; “*de novo* protein folding”, “translational frameshifting”, “positive regulation of autophagy”, and “regulation of autophagosome assembly” (Fig. 1*C*). In addition, “negative regulation of chromatin silencing at rDNA” has previously been shown to be important to restrict protein aggregation (34). Likewise, correct processing of mRNA is also predicted to affect protein aggregation, and the GO groups “nuclear-transcribed mRNA catabolic process” as well as “defense response to virus” came out as enriched processes in the screen: That “defense response to virus” was an enriched GO group was primarily due to three genes, *SKI2*, *SKI3*, and *SKI8*, that encode proteins of the SKI complex mediating 3′-5′ RNA degradation by the cytoplasmic exosome (37, 38).

The other signaling pathway identified as being important for restricting aggregation, apart from the ribosomal DNA silencing one, was the mTOR pathway (Fig. 1*C*), which is interesting as this pathway has been identified as a conserved pathway controlling the rate of aging (6, 39). We confirmed the respective mTOR-related screen hits (*NPR2*, *NPR3*, and *TCO89*) through manual strain constructions (Fig. 1*D*) and complementation analysis (Fig. S1, *A* and *B*). Interestingly, while the *TCO89* deletion (*tco89*Δ) in the screen demonstrated enhanced pro3-1 aggregation (Supplementary data 1), the confirmation experiment did not show any protein aggregation upon heat shock (Fig. 1*D*). This suggests the presence of an aggregation-enhancing suppressor mutation in the SGA deletion collection. A reduced aggregation propensity of *tco89*Δ upon heat shock (as found with the manually generated strain) is in line with Tco89 being a positive regulator of mTOR signaling. Consistent with this notion, the deletion of other positive regulators of mTOR signaling, mainly members of the SEA and EGO complex, also resulted in reduced pro3-1 aggregation (Fig. 1, *E* and *F*). An analysis of the fraction of pro3-1 in foci in relation to total cellular pro3-1 signal was mainly in line with aggregate counts (Fig. S1, *E* and *F*). In agreement with a previous study (40), we indeed found that *tco89*Δ cells have decreased mTOR signaling activity, while *npr3*Δ cells exhibit increased mTOR signaling activity (Fig. S1*D*). It is important to recognize that the number of pro3-1 foci during heat stress results from both protein aggregation and the coalescence of these aggregates. Consequently, distinguishing the specific effects of a mutant on either process can be challenging. However, based on a time series analysis, we propose that the mutants studied here predominantly influence the protein aggregation of pro3-1, because they exhibit a reduction of pro3-1 aggregates with time, indicating still functioning aggregate coalescence (Fig. S1*C*). Furthermore, considering the known roles of the studied genes in the mTOR pathway and protein translation, we suggest that protein aggregation is the primary impact of these mutants.

**Figure 2:**
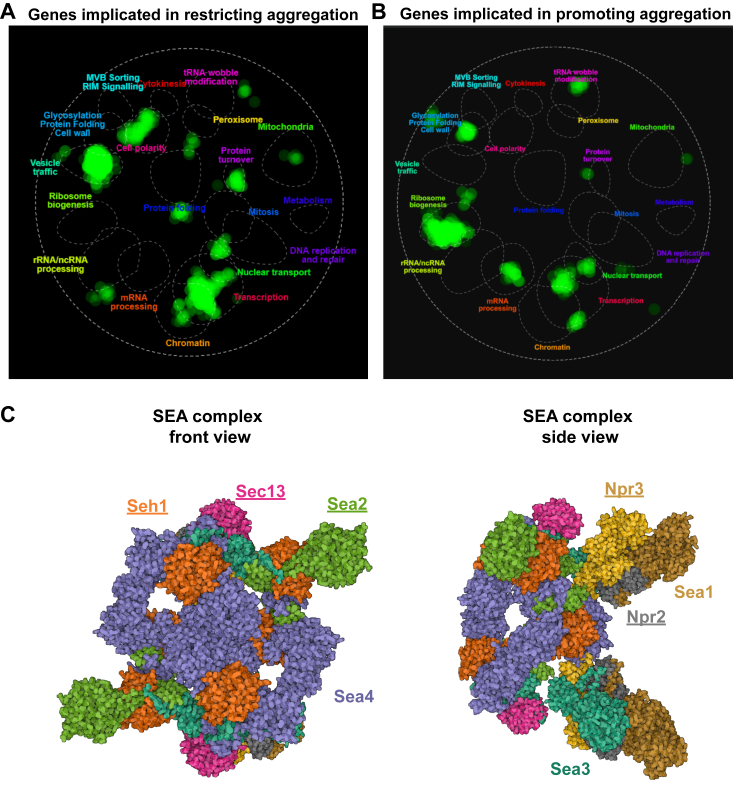
**Modifiers of pro3-1 protein aggregation, visualized by SAFE** with www.thecellmap.org identifying network regions enriched for similar GO biological process terms as outlined by *dashed white lines*. Maps of the genetic landscape of a yeast cell, with genes affecting pro3-1 protein aggregation denoted in *green*. *A*, top 49 screen hits identifying genes that restrict protein aggregation (cutoff: More than 1.0 identified pro3-1 foci per cell). Screen data of 90 min 38 °C heat shock was used. Identified genes specifically cluster around functions related to protein folding, cytoskeleton organization, cell polarity, and vesicle trafficking, indicating that these cellular functions are major determinants of temporal and spatial quality control. *B*, top 125 screen hits identifying genes that promote protein aggregation (cutoff: Less than 1.0 identified pro3-1 foci per cell). Screen data of 45 min 38 °C heat shock was used. Identified genes specifically cluster around functions related to ribosome biogenesis. *C*, consensus cryo-EM map of the SEA complex shown in front and side views (Image taken from the PDBe website. Accession code 8ADL). Underlined SEA members were shown to impact protein aggregation in this study (see also Supplementary data one and 2). *NPR2*, *NPR3*, and *SEC13* were hits in the screen for restricting pro3-1 protein aggregation. *SEA2* and *SEH1* were taken from the extended analysis for genes promoting pro3-1 protein aggregation from Fig. 1, *E* and *F*. GO, Gene Ontology; SAFE, spatial analysis of functional enrichment.

Other sets of genes identified by spatial analysis of functional enrichment (41, 42) as important in restricting protein aggregation included genes encoding proteins involved in vesicle trafficking, protein turnover, cell polarity, and transcription-related genes (Fig. 2*A*). Conversely, identifying genes promoting protein aggregation pinpointed predominantly ribosome biogenesis genes (Fig. 2*B*, Supplementary data 2, GO Biological process analysis shown in Fig. S2), which is interesting as mTOR activity is directly correlated to the overall activity of the protein synthesizing machinery. In this screen, we focused on the lower end of the aggregate quantification table, unlike the initial screen. We opted for a 45-min heat shock duration instead of 90 min to reduce the time available for aggregate coalescence. This approach amplifies the effects of the mutants, making differences in aggregation reduction clearer.

The identification of nearly all SEA complex proteins (Fig. 2*C*) as affecting aggregation underscores its pivotal role in proteostasis. Intriguingly, the SEA complex shares similarity and components with nuclear pores. However, our understanding of their function in proteostasis remains limited and warrants further exploration. In line with this, our screening among nonessential genes identified the deletion of the nuclear pore protein gene *NUP42* (Supplementary data 1) as the most significant contributor to increased protein aggregation following a heat shock. Prior research has shown that nuclear pores play a crucial role in transporting protein aggregates (26) or heat shock mRNAs (43) between the cytosol and the nucleus. The SEA complex was discovered when it was found that the nucleoporin Seh1 was present at the vacuolar membrane and interacted with the previously identified mTOR regulators Npr2 and Npr3 (11, 44).

An earlier study reported that Tor1 regulates protein solubility in *S. cerevisiae* (45). Contrary to our findings, the authors observed an accumulation of SDS-insoluble protein aggregates following TORC1 inhibition. These could potentially be small insoluble oligomers, not detected by the Hsp104 system, which was used in our study to monitor protein aggregation. Moreover, TORC1 inhibition has been shown to reverse cellular abnormalities and enhance mutant protein clearance in Hutchinson-Gilford Progeria syndrome cells (46), underscoring the evolutionary conserved role of mTOR in proteostasis.

### The aggregation of mTOR mutants is unlinked to overall protein synthesis and Hsf1 signaling

**Figure 3:**
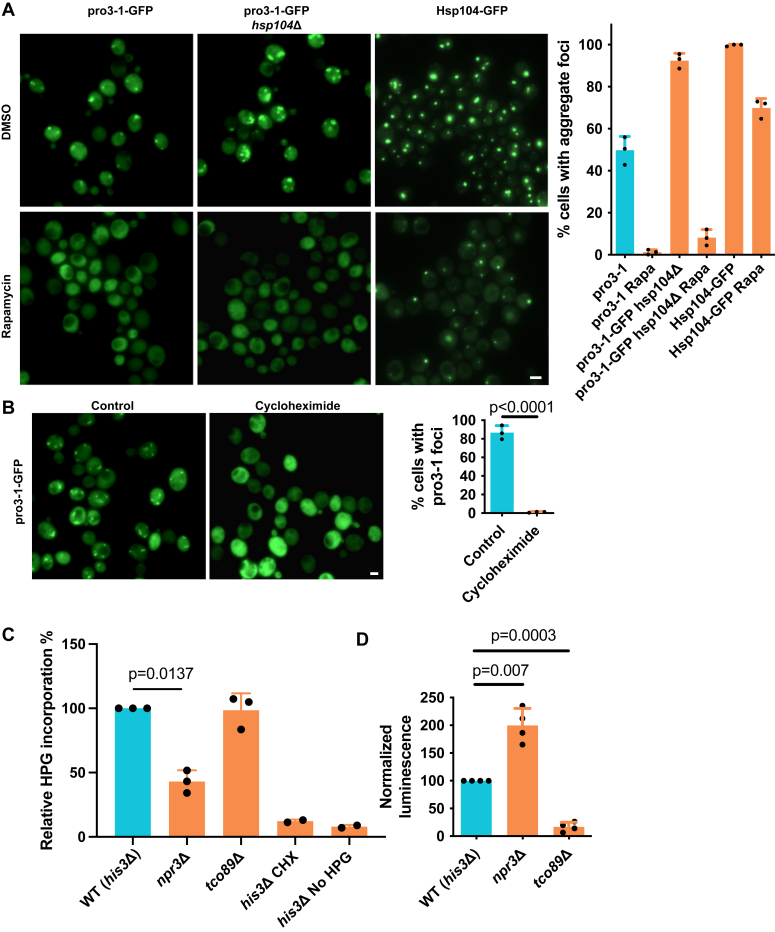
**Characterization of mTOR signaling pathway mutants in the context of protein aggregation.***A*, aggregation of pro3-1 and endogenous proteins during heat shock (HS) are dependent on mTOR signaling. Cells were pretreated for 30 min with 219 nM of the mTOR inhibitor rapamycin (Rapa), followed by a 90 min 38 °C HS. *Left*: representative fluorescence microscopy images of cells expressing pro3-1-GFP or Hsp104-GFP are shown. *Right*: quantification of cells with aggregate foci. Data are presented as mean values ± SD. *n* = 3 experiments. One-way ANOVA followed by Dunnett’s multiple comparison test. *B*, pro3-1 aggregation after HS is dependent on active protein translation. Cells were pretreated with 0.25 μg/ml cycloheximide (CHX) before HS. *Left*: representative fluorescence microscopy images of cells expressing pro3-1-GFP are shown. *Right*: quantification of cells with pro3-1 aggregates. Data are presented as mean values ± SD. *n* = 3 experiments. Unpaired *t* test (two-tailed). *C*, quantification of global translation rate in mTOR signaling pathway mutants. Data are presented as mean values ± SD. *n* = 3 independent experiments. One sample *t* test. Cycloheximide (CHX) and “No-HPG” controls were performed in *n* = 2 independent experiments. Signal was normalized to the *his3*Δ control. *D*, Hsf1 activity assay with mTOR pathway mutants. Luminescence was normalized to the *his3*Δ control. Data are presented as mean values ± SD. *n* = 3 independent experiments. One sample *t* test. Scale bars represent 2 μm. mTOR, mechanistic target of rapamycin; HPG, L-homopropargylglycine.

To further elucidate the role of mTOR signaling in protein aggregation, we subjected pro3-1-GFP expressing cells to treatment with the mTOR inhibitor rapamycin, followed by a heat shock. This intervention almost fully eliminated pro3-1 aggregation, even in an *hsp104*Δ background that has previously been shown to exhibit markedly increased protein misfolding (24, 47) (Fig. 3*A*). Additionally, general protein aggregation, as indicated by Hsp104-GFP, was significantly reduced with rapamycin (Fig. 3*A*). To investigate whether pro3-1 misfolds cotranslationally, we administered cycloheximide (CHX) to cells before the heat shock. This intervention significantly diminished pro3-1 aggregation (Fig. 3*B*), suggesting that active translation is a prerequisite for pro3-1 aggregation and that it is the newly synthesized pro3-1 that is aggregating during heat shock (at 38 °C). Alternatively, it might be possible that CHX is reducing pro3-1 foci by preventing translation of some factor required for assembly of pro3-1 into foci. Combined with our above results with rapamycin, this indicates that rapamycin may prevent pro3-1 aggregation by inhibiting protein translation as rapamycin was previously demonstrated to reduce translation rates (48). However, when we assessed global translation rates *via* incorporation of the methionine analog L-homopropargylglycine (HPG) in newly synthesized proteins, we found, unexpectedly, that the translation rate of the *tco89*Δ mutant was not different from the control (Fig. 3*C*). Furthermore, the *npr3*Δ mutant exhibited a reduced translation rate (Fig. 3*C*), potentially an adaptation to the proteostatic stress described above under steady-state conditions. Thus, altered aggregation observed in the mTOR mutants analyzed cannot be explained simply by effects on overall protein synthesis. Thus, mTOR-affected protein aggregation may be regulated by translation-independent mechanisms potentially involving chaperones or other PQC machineries. Indeed, recent data have shown that TORC1 inhibition induces 19S regulatory particle assembly-chaperones as well as proteasome subunits (49). Also, as mentioned above, reduced aggregation achieved by rapamycin appears to be independent of the presence of the disaggregase Hsp104.

To investigate whether mTOR affected protein aggregation through effects on the heat shock response, we measured the activity of a Hsf1-dependent stress response reporter (50) in mTOR mutants. The *npr3Δ* mutant displayed elevated Hsf1 activity (Fig. 3*D*), while the *tco89*Δ mutant exhibited decreased Hsf1 activity compared to the control, suggesting that reduced mTOR activity inhibits protein aggregation in the absence of an Hsf1-dependent stress response. Otherwise, activated Hsf1 and consequently inducing expression of heat shock genes would have been responsible for lowering protein aggregation, which was not the case in *tco89Δ* cells.

### Reducing mTOR activity affects aggregation in young and old cells differently

**Figure 4:**
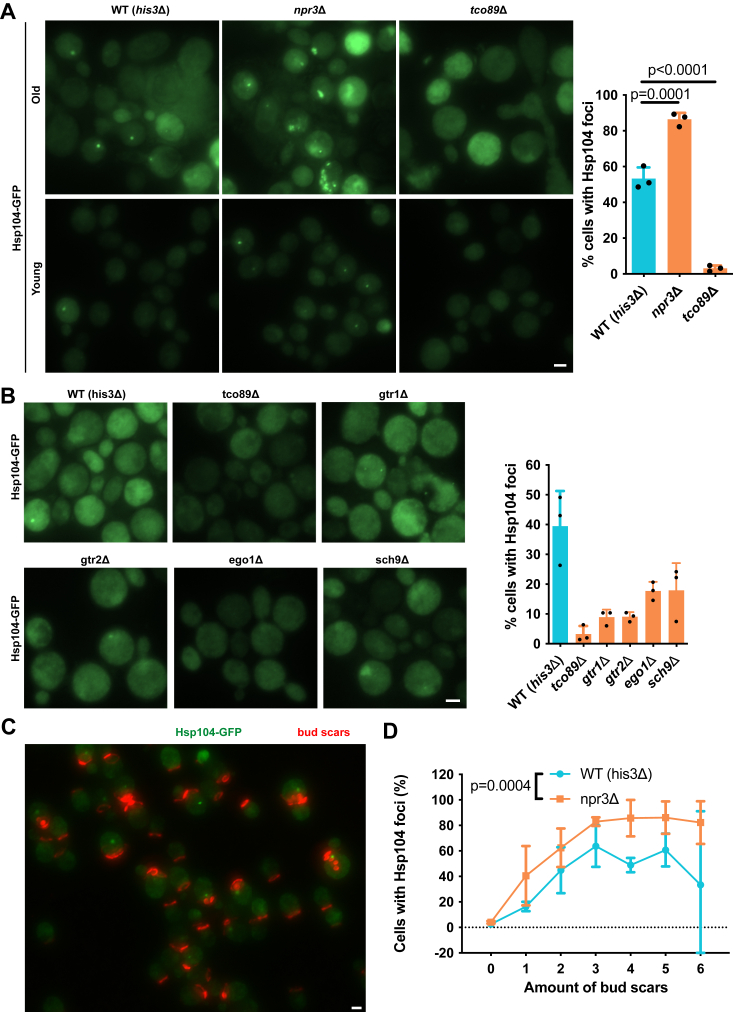
**Protein aggregation of mTOR pathway mutants during replicative aging.***A*, protein aggregation during replicative aging in mTOR signaling mutants. Median age of old cells is about 12 cell divisions. *Left*: representative fluorescence microscopy images of cells expressing Hsp104-GFP. *Right*: quantification of the percentage of old cells with Hsp104 foci. Data are presented as mean values ± SD. *n* = 3 independent experiments. One-way ANOVA followed by Dunnett’s multiple comparison test. *B*, protein aggregation during replicative aging in mutants of positive mTOR regulators. *Left*: representative fluorescence microscopy images of cells expressing Hsp104-GFP. *Right*: quantification of the percentage of cells with Hsp104 foci. Data are presented as mean values ± SD. n = 3 independent experiments. *C*, representative fluorescence microscopy images of Hsp104 inclusions during first cell divisions in *npr3Δ* cells. Cells were expressing Hsp104-GFP (shown in *green*). Bud scars were stained with WGA-Alexa594 and shown in *red*. *D*, quantification of cells with Hsp104 inclusions during first cell divisions in *npr3Δ* cells from C. Data are presented as mean values ± SD. *n* = 3 independent experiments. One-way ANOVA. Scale bars represent 2 μm. mTOR, mechanistic target of rapamycin.

When conducting old cell isolations we observed that old *npr3*Δ cells exhibited more Hsp104-associated protein aggregates than their WT counterparts (Fig. 4*A*). Remarkably, old *tco89*Δ cells displayed almost no Hsp104 foci. These differences between WT and *tco89*Δ cells, albeit less pronounced, were also discernible in young cells. Other mTOR pathway deletion mutants also showed reduced protein aggregation during aging, although not as low as in *tco89*Δ cells (Fig. 4*B*). These findings demonstrate that young npr3Δ cells already display a significant number of cells with Hsp104 inclusions, even in the absence of external stressors.

To determine the time point at which *npr3*Δ cells begin to exhibit Hsp104 inclusions during their lifespan, we conducted a bud scar staining analysis. At each generational stage, *npr3*Δ mother cells demonstrated a higher proportion of cells with Hsp104 inclusions than WT (Fig. 4, *C* and *D*). Notably, 50% of cells positive for Hsp104 inclusions were reached at 1.5 or 2.4 divisions for *npr3*Δ and WT cells, respectively. We observed a plateau in the proportion of cells with Hsp104 foci after about three divisions for both npr3Δ and WT mother cells, which persisted until old age (Fig. 4*D*). These findings suggest that *npr3*Δ mother cells continuously generate more protein aggregates than WT throughout their lifespan.

**Figure 5:**
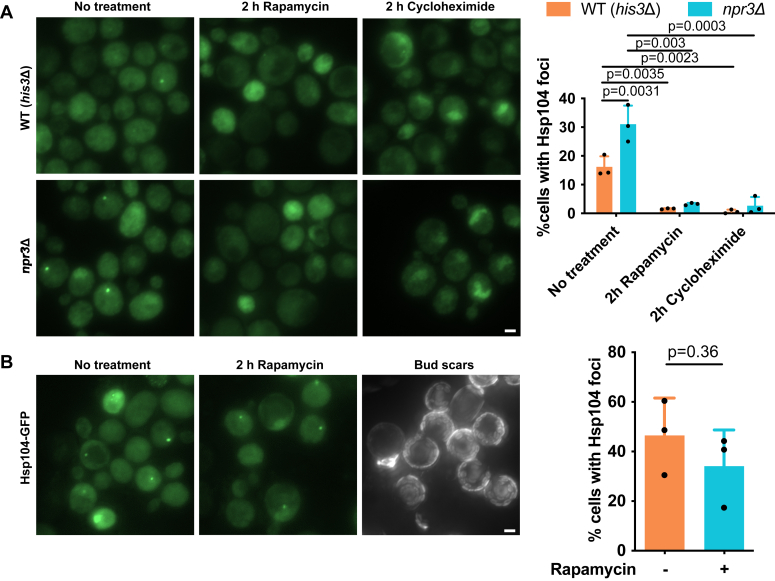
**Dynamics of the age-associated protein aggregate deposit.***A*, age-associated protein aggregate deposits are removable in yeast cells of younger age. Cells were treated in exponential phase for 2 h with either 219 nM rapamycin or 0.25 μg/ml cycloheximide. *Left*: representative fluorescence microscopy images of cells expressing Hsp104-GFP. *Right*: quantification of the percentage of cells with Hsp104 foci. Data are presented as mean values ± SD. *n* = 3 independent experiments. One-way ANOVA followed by Dunnett’s multiple comparison test. *B*, age-associated protein deposits are less removable in old yeast cells. Old yeast cells (median age about 12 divisions) were treated for 2 h with 219 nM rapamycin. *Left*: representative fluorescence microscopy images of cells expressing Hsp104-GFP. *Right*: quantification of the percentage of cells with Hsp104 foci. Data are presented as mean values ± SD. *n* = 3 independent experiments. Unpaired *t* test (two-tailed): n.s., not significant.

To test if the Hsp104-associated aggregate deposits could be eliminated in young *npr3*Δ cells taken from a log-phase culture by reducing mTOR signaling and/or reducing translation, we treated them with rapamycin or CHX. With both drugs, the inclusions vanished in both WT and *npr3*Δ cells, indicating they are dependent on mTOR signaling and active protein translation (Fig. 5*A*). However, when we subjected replicative old yeast cells to rapamycin treatment, the age-associated Hsp104 inclusions remained intact (Fig. 5*B*), indicating that those inclusions may harbor more terminally misfolded aggregates. This aligns with a previous study, suggesting that the contents of the age-associated Hsp104-marked inclusions in old cells may be amyloid-like (51). This protein inclusion was termed Agepod previously (51) and its origin remains unclear. However, the fact that the generation of these inclusions could be almost totally abolished by deleting *TCO89* suggests their formation during aging is somehow controlled by mTOR signaling.

### mTOR signaling affects sensitivity to mutant huntingtin

**Figure 6:**
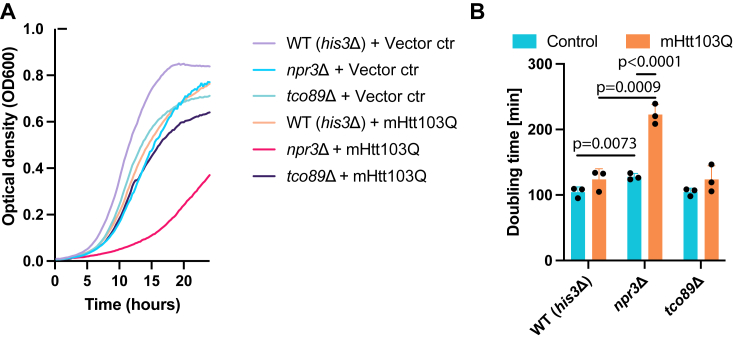
**mTOR signaling affects sensitivity to mutant Huntingtin.***A*, growth curves of mTOR pathway mutants expressing constitutively mHtt103Q. *B*, quantification of cell division time from *A*. Data are presented as mean values ± SD. *n* = 3 independent experiments. One-way ANOVA followed by Dunnett’s multiple comparison test. mTOR, mechanistic target of rapamycin; mHtt, mutant Huntingtin.

To evaluate whether the effect of mTOR mutants on protein aggregation transcended to additional proteostatic stress, we expressed human polyQ-expanded mutant Huntingtin (mHtt), the causal agent of Huntington’s disease, and analyzed the fitness of the mutants by growth rate analysis. We found that *npr3*Δ cells displayed severe fitness defects when expressing mHtt; a 74.2% increase in division time. WT cells showed an 18.6% and *tco89*Δ cells showed an 18.4% increase in division time after mHtt expression (Fig. 6, *A* and *B*). Without mHtt expression, the *npr3*Δ mutant showed a 23% increase in cell division time compared to WT. The data indicate that mTOR signaling is closely associated with proteostasis and proteotoxicity, suggesting that hyperactive TOR signaling imposes stress on cells. In conclusion, our work demonstrates that mTOR signaling and, in particular, the SEA complex are key factors in age-associated and heat shock-induced protein aggregation.

## Experimental procedures

### Strains, plasmids, and growth conditions

Plasmids and strains are listed in Tables S1 and S2, respectively. The strains used in this study are derivatives of BY4741 (*MATa his3Δ1 leu2Δ0 met15Δ0 ura3Δ0*). Cells were cultured at 30 °C in rich YPD medium, in complete synthetic medium or in synthetic dropout media (Formedium). Deletion strains were from the Yeast Knockout Collection (52) and the gene deletions were confirmed by PCR (Fig. S3). Strains with GFP-labeled endogenous proteins were from the yeast GFP clone collection (Thermo Fisher Scientific) (53). All plasmids were verified by DNA sequencing. Rapamycin treatment was performed 30 min prior to other treatments and with a final concentration of 219 nM (200 ng/ml). Rapamycin was initially dissolved in dimethyl sulfoxide. CHX was used at 250 μg/ml with 30 min of pretreatment. CHX was initially dissolved in ethanol.

### Strain construction

Transformations were done using the standard lithium acetate protocol, and transformants were confirmed using PCR and phenotypic assays.

### Growth time analysis

Growth time analysis was performed as previously described (54). Briefly, yeast cells were grown overnight at 30 °C, diluted to an absorbance at 600 nm (*A*_600_) of 0.1 and distributed in a 24-well microtiter plate with a total volume of 800 μl per well. The microtiter plate was then sealed with a MicroAmp optical adhesive film (Thermo Fisher Scientific) and punctured with a 23 Gauge needle for air exchange. A plate reader (BioTek Synergy 2 SL Microplate Reader Luminescence and Biotek Gen5 Data Analysis Software, www.agilent.com) was used to monitor *A*_600_ of the cultures every 15 min for 24 h.

### Old cell isolation

Old cells were obtained using the magnetic beads biotin-streptavidin system according to established protocols (55, 56). Cells were grown to exponential phase at 30 °C, one A_600_ unit of cells (approximately 3 × 10^7^ cells) were harvested, washed in PBS and then labeled with EZ link Sulfo-NHS-LC biotin (Thermo Fisher Scientific) at a final concentration of 0.5 mg/ml in 1 ml PBS. Excess biotin was washed away, and cells were resuspended in 1 L growth medium and cultured for about 15 h overnight at 30 °C, using an orbital shaker (180 rpm). When the cultures had reached an *A*_600_ of about 0.5, cells were washed with PBS and incubated with 0.05 mg/ml MagnaBind streptavidin beads (Thermo Fisher Scientific). Biotin-labeled cells were then isolated using a magnetic sorter, followed by three washes with PBS containing 0.5% glucose. Cells were resuspended in 7 ml growth medium and used directly for subsequent experiments. Median age of the old cells was determined by counting bud scars in z-stack images upon staining cells with 10 μg/ml Wheat Germ Agglutinin (Thermo Fisher Scientific).

### Phospho-RPS6 detection by Western blot

Phospho-RPS6 detection by Western blot was done as previously described (57). Briefly, cells were precultured overnight, diluted to *A*_600_ = 0.1 and grown until midexponential phase (*A*_600_∼0.8) in YPD at 30 °C. Cells were then treated with or without rapamycin for 30 min at a final concentration of 219 nM (200 ng/ml). Cells were harvested as eight *A*_600_ units, resuspended in 100 μl of lysis buffer (50 mM Tris–HCl pH 7.5, 50 mM NaCl, 15% glycerol, 0.5% Tween-20, 1 mM PMSF, and EDTA-free Roche complete protease inhibitor cocktail) and 150 μl glass beads were added. Lysis of cells was performed in a bead beater using five cycles of 45 s breaking, 5.5 speed + 3 min on ice. Protein concentration of the cleared lysate was determined by the Bradford assay. Lyzed cells were resuspended in Laemmli sample buffer (Bio-Rad) supplemented with β-mercaptoethanol and boiled for 5 min at 95 °C. Subsequently, 10 μl was typically loaded per lane on a 4 to 15% precast polyacrylamide 18 well Criterion TGX protein gel (Bio-Rad). Gel transfer onto polyvinylidene fluoride membranes was performed using a semi-dry blotting system (Criterion, Bio-Rad). Membranes were probed with phospho-Ser235/Ser236-S6 (#2211, Cell Signaling Technology) and anti-Pgk1 (ab90787, Abcam, 1/10,000 dilution) as a loading control overnight at 4 °C. As secondary antibodies, goat anti-mouse IRDye 800CW and goat anti-rabbit IRDye 680RD were used (LI-COR; 1/20,000 dilution) and membranes were scanned using the LI-COR Odyssey Infrared scanner. Protein levels were calculated by measuring the background corrected band intensities using Fiji/ImageJ software (https://imagej.net/software/fiji/) and normalizing to Pgk1 protein levels.

### Fluorescence microscopy

Fluorescence microscopy was performed as previously described (54). Briefly, images were mostly obtained using an Axio Observer Z1 ﬂuorescence microscope (Zeiss), equipped with an Axiocam 506 mono camera and a 20x, as well as a 100x oil objective lens (Plan-APOCHROMAT 100x/1.4 Oil DIC) controlled by the Zen blue software (www.zeiss.com). Raw data were collected as Z-stacks and projected using ImageJ (NIH) with manual quantification.

### Heat shock treatments

Yeast cells were subjected to a continuous 38 °C heat shock in a shaking water bath, using 100 ml Erlenmeyer flasks.

### Hsf1 activity reporter assay

The activity of the Hsf1-dependent stress response was determined as described previously (50). Briefly, yeast cells were transformed with a plasmid (pAM10) carrying the luciferase NanoLuc under the control of a heat shock promoter (HSE) or with a control plasmid (pAM09). Nano-Glo substrate (Promega) was diluted 1:100 with the supplied lysis buffer and mixed 1:10 with cells grown in SD-Ura medium in a white 96-well plate. Bioluminescence was determined immediately, using a plate reader (Biotek Synergy 2 SL).

### Synthetic genetic array screen and high content microscopy

A genome-wide SGA screen was performed as described previously (58), using a BM3-BC colony handling robot (S&P Robotics Inc). The screen was run in 1536 spot format with the pro3-1-mCherry query strain (with GFP-labeled endogenous Hsp104) in the Y7092 background, crossed to the Yeast Knockout Collection (52) or the temperature-sensitive (ts) mutant array in essential genes (59). Final strains were grown in 96 well plates in liquid SD medium to an *A*_600_ of about 0.5. Cells were then subjected to a 38 °C heat shock for 45 min (screen for genes promoting pro3-1 aggregation) or 90 min (screen for genes restricting pro3-1 aggregation) in a water bath using the KO collection. For the temperature-sensitive mutant array in essential genes, cells were subjected to a 38 °C heat shock for 60 min (screen for genes promoting pro3-1 aggregation) or 110 min (screen for genes restricting pro3-1 aggregation). The cells were then fixed with 3.7% formaldehyde for 30 min and washed in PBS. Next, the cells were transferred to a black 96 well plate with a glass bottom (Matriplate). The screen was performed once. Imaging was then performed with an ImageXpress Micro high content microscope (Molecular Devices). Hsp104-GFP and pro3-1-mCherry foci were identified using the MetaXpress software (Molecular Devices, www.moleculardevices.com). Their amount per cell and fluorescence intensity was measured, as well as other parameters (fluorescence intensity of the whole cell and colocalization between Hsp104-GFP and pro3-1-mCherry foci). For the screen for identifying genes promoting pro3-1 protein aggregation a heat shock duration of 90 min was performed and the 49 top hits were selected (cutoff: More than 1.0 identified pro3-1 inclusions per cell). For the screen for identifying genes promoting pro3-1 protein aggregation a heat shock duration of 45 min was chosen to increase screen resolution and the top 125 hits were selected (cutoff: Less than 1.0 identified pro3-1 inclusions per cell). GO analysis of biological process enrichment was performed with the DAVID Functional Annotation Tool (david.ncifcrf.gov), using the GOTERM_BP_DIRECT option. For all GO analyses, default settings were used, and categories with a *p*-value of < 0.05 were considered as signiﬁcantly enriched. Only enriched pathways with at least three screen hit genes were considered.

### Translation assay

For determining the global translation rate, the Click-iT HPG Alexa Fluor 488 Protein Synthesis Assay Kit (Thermo Fisher Scientific) was used. Briefly, 1 × 10^7^ cells (Absorbance = 0.45) were used. After centrifugation at 5000 rpm for 5 min, the cell pellet was resuspended in 1 ml HC-Met media. In addition, 50 μM of HPG was added. For the negative control, 35 μg/ml CHX was used. For the blank control, HPG and CHX were omitted. Cells were incubated for 30 min at 200 rpm and 30 °C, followed by a wash with 0.5 ml PBS. Subsequently, 500 μl of ice-cold 70% ethanol (−20 °C) was added followed by vortexing to fix cells. Cells were washed with 0.5 ml PBS, followed by addition of 0.5 ml of 1% Triton X-100 in PBS and incubated for 20 min at room temperature to permeabilize cells. Cells were then washed with 0.5 ml 1% bovine serum albumin in PBS. Then, 0.5 ml of click-iT reaction cocktail was added and incubated for 30 min in dark. Cells were washed with 0.5 ml of 1% bovine serum albumin in PBS and resuspended in 1 ml PBS. The cells were transferred to a black 96-well plate with a glass bottom (MatriPlate). Imaging was then performed with an ImageXpress Micro high content microscope (Molecular Devices) to determine fluorescence intensities.

### Automated single-cell quantification of fraction of pro3-1 in foci

For determining the fraction of pro3-1 in foci, fluorescence microscopy images were quantified using Zeiss Zen 3.7 software (www.zeiss.com). The images were taken from cells expressing pro3-1-GFP (from Fig. 1, *D* and *E*). To segment cells, a global thresholding was used together with “Otsu Threshold (Light Regions)” as method setting. A minimum object size of 500 pixels was defined. To identify pro3-1 foci, a segmentation with background subtraction was performed using the “Rolling Ball” option with a radius of 3. Additionally, the “Otsu Threshold (Light Regions)” method setting was applied. Intensity sum values of the pixels were calculated and displayed. The pro3-1 intensity sum of the pixels in the aggregates of one cell was divided by the pro3-1 intensity sum of all pixels in the respective cell. For percentage values, a multiplication by 100 was performed. In this way, the fraction of pro3-1 in foci in relation to the total pro3-1 signal per cell was determined. All analyzed cells from the three independent experiments were pooled and sorted according to fraction value yielding a profile, which was plotted using GraphPad Prism 9 (www.graphpad.com). For all different strains, the same number of cells was analyzed for optimal comparability.

### Statistical analysis

Data collection and date quantifications were performed with Microsoft Excel 2016. All attempts to repeat the experiments were successful. Quantification and statistical analysis were performed using GraphPad Prism 9. Data in graphs are presented as mean ± SD and were analyzed using one-way ANOVA followed by Dunnett’s multiple comparison test, or unpaired *t* test (two-tailed) if not stated otherwise. The experiments were performed in three independent experiments unless specified.

## Data availability

All data needed to evaluate the conclusions in the paper are present in the paper and/or the Supplementary Materials. Source data are provided with this paper.

## Supporting information

This article contains supporting information.

## Declaration of generative AI and AI-assisted technologies in the writing process

During the preparation of this work, authors used ChatGPT (GPT-3 and GPT-4, OpenAI’s large-scale language-generation model) to improve the writing style of this article. After using this tool/service, the authors reviewed and edited the content as needed and take full responsibility for the content of the publication.

## Conflict of interest

The authors declare that they have no conflicts of interest with the contents of this article.

## Supporting information

Supplemetary Data 1

Supplemetary Data 2
