# Supplementary material for: mTOR signaling controls protein aggregation during heat stress and cellular aging in a translation- and Hsf1-independent manner
Source: J Biol Chem. 2025 Jan 10;301(2):108172. doi: 10.1016/j.jbc.2025.108172 (PMC11849620; doi:10.1016/j.jbc.2025.108172)
Supplement: Supplementary Tables 241116 [file mmc3.pdf]

Table S1. Plasmid list.

Plasmid	Replicon	Promoter	Gene	Backbone	Parent	Selection marker	Reference
p416 103Q GPD	CEN/ARS	GPD	mHtt103Q-GFP	pRS416	p416 GPD	URA3	Krobitsch, Lindquist (1)
pAM09	CEN/ARS	-	-			URA3	Masser, Kandasamy, Kaimal, Andréasson (2)
pAM10	CEN/ARS	CYC1–HSE	NanoLuc			URA3	Masser, Kandasamy, Kaimal, Andréasson (2)
pAF002	-	GPD	GFP-GPDp-pro3-1-mCherry	pRS403		NAT	This study
pAF033	-	-	GPDp-pro3-1-GFP with MET15 homology regions	pRS303	pPW411	NAT	This study
pAF055	-	GPD	TCO89p-TCO89-TCO89t	pRS403		HIS3	This study
pAF056	-	GPD	NPR3p-NPR3-NPR3t	pRS403		HIS3	This study
pPW351	-	GPD	pro3-1-GFP	pRS403		HIS3	Schneider, Nystrom, Widlund (3)
pPW411	-	-	MET15 homology regions	pRS303		NAT	Fischbach, Johns, Schneider, Hao, Tessarz, Nyström (4)
pRS416	CEN/ARS	-	-	pRS416		URA3	Sikorski, Hieter (5)
pYM22	-	-	GFP			HIS3	Janke, Magiera, Rathfelder, Taxis, Reber, Maekawa, Moreno-Borchart, Doenges, Schwob, Schiebel, Knop (6)
Htt103Q-mCherry	CEN/ARS	GPD	GPDp-mHtt103Q-mCherry	pRS416	p416 103Q GPD	URA3	Fischbach, Johns, Schneider, Hao, Tessarz, Nyström (4)

Table S2. Yeast strain list.

ID	Name	Genotype	Parent strain	Ingegrated plasmid	Reference
BY4741	BY4741	MATa his3Δ1 leu2Δ0 met15Δ0 ura3Δ0			
yAF002	Hsp104-GFP-GPD-pro3-1-mCherry query strain	*MATα can1Δ::STE2pr-Sp_his5 lyp1Δ his3Δ1 leu2Δ0 ura3Δ0 met15Δ0 LYS2+ Hsp104-GFP-pGPD-pro3-1-mCherry-NatMX*	Y7092	pAF002	This study
BY4741	BY4741	*MATa his3Δ1 leu2Δ0 met15Δ0 ura3Δ0*			
Y7092	Y7092	*MATα can1Δ::STE2pr-Sp_his5 lyp1Δ his3Δ1 leu2Δ0 ura3Δ0 met15Δ0 LYS2+*			Tong, Evangelista, Parsons, Xu, Bader, Page, Robinson, Raghibizadeh, Hogue, Bussey, Andrews, Tyers, Boone (7)
hsp104Δ	hsp104Δ	***MATa** Hsp104Δ::kanMX4 his3Δ1 leu2Δ0 met15Δ0 ura3Δ0*			Deletion collection (8)
Hsp104-GFP	Hsp104-GFP	***MATa** his3Δ1 leu2Δ0 met15Δ0 ura3Δ0 HSP104-GFP-HIS3MX6*	BY4741		GFP collection (9)
yAF021	hsp104Δ + pro3-1-GFP	***MATa** Hsp104Δ::kanMX4 his3Δ1::HIS3-pGPD-pro3-1-GFP-tPgk1 leu2Δ0 met15Δ0 ura3Δ0*	hsp104Δ	pPW351	This study
PWY1180	pro3-1-GFP	***MATa** Hsp104Δ::kanMX4 his3Δ1::HIS3-pGPD-pro3-1-GFP-tPgk1 leu2Δ0 met15Δ0 ura3Δ0*	BY4741		Schneider, Nystrom, Widlund (3)
npr3Δ	npr3Δ	***MATa** npr3Δ::kanMX4 his3Δ1 leu2Δ0 met15Δ0 ura3Δ0*	BY4741		Deletion collection (8)
tco89Δ	tco89Δ	***MATa** tco89Δ::kanMX4 his3Δ1 leu2Δ0 met15Δ0 ura3Δ0*	BY4741		Deletion collection (8)
his3Δ	his3Δ	***MATa** his3Δ::kanMX4 his3Δ1 leu2Δ0 met15Δ0 ura3Δ0*	BY4741		Deletion collection (8)
yAF107	npr2Δ + pro3-1-GFP	*MATa his3Δ1 leu2Δ0 ura3Δ0 met15Δ::GPDp-pro3-1-GFP-PGK1t-NatMX npr2Δ::kanMX4*	BY4741	pAF033	This study
yAF108	npr3Δ + pro3-1-GFP	*MATa his3Δ1 leu2Δ0 ura3Δ0 met15Δ::GPDp-pro3-1-GFP-PGK1t-NatMX npr3Δ::kanMX4*	BY4741	pAF033	This study
yAF109	tco89Δ + pro3-1-GFP	*MATa his3Δ1 leu2Δ0 ura3Δ0 met15Δ::GPDp-pro3-1-GFP-PGK1t-NatMX tco89Δ::kanMX4*	BY4741	pAF033	This study
yAF110	his3Δ + pro3-1-GFP	*MATa his3Δ1 leu2Δ0 ura3Δ0 met15Δ::GPDp-pro3-1-GFP-PGK1t-NatMX his3Δ::kanMX4*	BY4741	pAF033	This study
yAF124	npr3Δ + Hsp104-GFP	*MATa his3Δ1 leu2Δ0 ura3Δ0 met15Δ0 npr3Δ::kanMX4 HSP104-GFP-Sp_HIS5*	npr3Δ	PCR pYM28	This study
yAF125	tco89Δ + Hsp104-GFP	*MATa his3Δ1 leu2Δ0 ura3Δ0 met15Δ0 tco89Δ::kanMX4 HSP104-GFP-Sp_HIS5*	tco89Δ	PCR pYM28	This study
yAF126	his3Δ + Hsp104-GFP	*MATa his3Δ1 leu2Δ0 ura3Δ0 met15Δ0 his3Δ::kanMX4 HSP104-GFP-Sp_HIS5*	BY4741	PCR pYM28	This study

tor1Δ	tor1Δ	*MAT**a** tor1Δ::kanMX4 his3Δ1 leu2Δ0 met15Δ0 ura3Δ0*	BY4741		Deletion collection (8)
sea2Δ	sea2Δ	*MAT**a** sea2Δ::kanMX4 his3Δ1 leu2Δ0 met15Δ0 ura3Δ0*	BY4741		Deletion collection (8)
gtr1Δ	gtr1Δ	*MAT**a** gtr1Δ::kanMX4 his3Δ1 leu2Δ0 met15Δ0 ura3Δ0*	BY4741		Deletion collection (8)
gtr2Δ	gtr2Δ	*MAT**a** gtr2Δ::kanMX4 his3Δ1 leu2Δ0 met15Δ0 ura3Δ0*	BY4741		Deletion collection (8)
ego1Δ	ego1Δ	*MAT**a** ego1Δ::kanMX4 his3Δ1 leu2Δ0 met15Δ0 ura3Δ0*	BY4741		Deletion collection (8)
yAF146	tor1Δ + pro3-1-GFP	*MAT**a** tor1Δ::kanMX4 his3Δ1 leu2Δ0 ura3Δ0 met15Δ0::GPDp-pro3-1-GFP-PGK1t-NatMX*	tor1Δ	pAF033	This study
yAF147	sea2Δ + pro3-1-GFP	*MAT**a** sea2Δ::kanMX4 his3Δ1 leu2Δ0 ura3Δ0 met15Δ0::GPDp-pro3-1-GFP-PGK1t-NatMX*	sea2Δ	pAF033	This study
yAF149	gtr1Δ + pro3-1-GFP	*MAT**a** gtr1Δ::kanMX4 his3Δ1 leu2Δ0 ura3Δ0 met15Δ0::GPDp-pro3-1-GFP-PGK1t-NatMX*	gtr1Δ	pAF033	This study
yAF150	gtr2Δ + pro3-1-GFP	*MAT**a** gtr2Δ::kanMX4 his3Δ1 leu2Δ0 ura3Δ0 met15Δ0::GPDp-pro3-1-GFP-PGK1t-NatMX*	gtr2Δ	pAF033	This study
yAF151	ego1Δ + pro3-1-GFP	*MAT**a** ego1Δ::kanMX4 his3Δ1 leu2Δ0 ura3Δ0 met15Δ0::GPDp-pro3-1-GFP-PGK1t-NatMX*	ego1Δ	pAF033	This study
seh1Δ	seh1Δ (YGL100WΔ)	*MAT**a** seh1Δ::kanMX4 his3Δ1 leu2Δ0 met15Δ0 ura3Δ0*	BY4741		Euroscarf
sch9Δ	sch9Δ (YHR205WΔ)	*MAT**a** sch9Δ::kanMX4 his3Δ1 leu2Δ0 met15Δ0 ura3Δ0*	BY4741		Euroscarf
yAF164	seh1Δ + pro3-1-GFP	*MAT**a** seh1Δ::kanMX4 his3Δ1 leu2Δ0 ura3Δ0 met15Δ0::GPDp-pro3-1-GFP-PGK1t-NatMX*	seh1Δ	pAF033	This study
yAF174	gtr1Δ + Hsp104-GFP	*MATa his3Δ1 leu2Δ0 ura3Δ0 met15Δ0 gtr1Δ::kanMX4 HSP104-GFP-Sp_HIS5*	gtr1Δ	PCR pYM28p	This study
yAF175	gtr2Δ + Hsp104-GFP	*MATa his3Δ1 leu2Δ0 ura3Δ0 met15Δ0 gtr2Δ::kanMX4 HSP104-GFP-Sp_HIS5*	gtr2Δ	PCR pYM28	This study
yAF176	ego1Δ + Hsp104-GFP	*MATa his3Δ1 leu2Δ0 ura3Δ0 met15Δ0 ego1Δ::kanMX4 HSP104-GFP-Sp_HIS5*	ego1Δ	PCR pYM28	This study
yAF177	seh1Δ + Hsp104-GFP	*MATa his3Δ1 leu2Δ0 ura3Δ0 met15Δ0 seh1Δ::kanMX4 HSP104-GFP-Sp_HIS5*	seh1Δ	PCR pYM28	This study
yAF178	sch9Δ + Hsp104-GFP	*MATa his3Δ1 leu2Δ0 ura3Δ0 met15Δ0 sch9Δ::kanMX4 HSP104-GFP-Sp_HIS5*	sch9Δ	PCR pYM28	This study
yAF165	sch9Δ + pro3-1-GFP	*MAT**a** sch9Δ::kanMX4 his3Δ1 leu2Δ0 ura3Δ0 met15Δ0::GPDp-pro3-1-GFP-PGK1t-NatMX*	sch9Δ	pAF033	This study
yAF179	npr3Δ + NPR3	*MATa leu2Δ0 ura3Δ0 met15Δ0 npr3Δ::kanMX4 his3Δ1::NPR3p-NPR3-NPR3t-HIS3*	npr3Δ	pAF056	This study
yAF180	npr3Δ + empty vector	*MATa leu2Δ0 ura3Δ0 met15Δ0 npr3Δ::kanMX4 his3Δ1::pRS403(HIS3)*	npr3Δ	pRS403	This study
yAF181	tco89Δ + TCO89	*MATa his3Δ1 leu2Δ0 ura3Δ0 met15Δ0 tco89Δ::kanMX4 his3Δ1::TCO89p-TCO89-TCO89t-HIS3*	tco89Δ	pAF055	This study

yAF182	tco89Δ + empty vector	*MATa leu2Δ0 ura3Δ0 met15Δ0 tco89Δ::kanMX4 his3Δ1::pRS403(HIS3)*	tco89Δ	pRS403	This study
yAF244	npr3Δ + pAM09	*MATa his3Δ1 leu2Δ0 ura3Δ0 met15Δ0 npr3Δ::kanMX4 pAM09(URA)*	npr3Δ		This study
yAF245	npr3Δ + pAM10	*MATa his3Δ1 leu2Δ0 ura3Δ0 met15Δ0 npr3Δ::kanMX4 pAM10(URA)*	npr3Δ		This study
yAF246	tco89Δ + pAM09	*MATa his3Δ1 leu2Δ0 ura3Δ0 met15Δ0 tco89Δ::kanMX4 pAM09(URA)*	tco89Δ		This study
yAF247	tco89Δ + pAM10	*MATa his3Δ1 leu2Δ0 ura3Δ0 met15Δ0 tco89Δ::kanMX4 pAM10(URA)*	tco89Δ		This study
yAF248	his3Δ + pAM09	*MATa his3Δ1 leu2Δ0 ura3Δ0 met15Δ0 his3Δ::kanMX4 pAM09(URA)*	his3Δ		This study
yAF249	his3Δ + pAM10	*MATa his3Δ1 leu2Δ0 ura3Δ0 met15Δ0 his3Δ::kanMX4 pAM10(URA)*	his3Δ		This study
yAF590	npr3Δ + Htt103Q-mCherry	*MATa his3Δ1 leu2Δ0 ura3Δ0 met15Δ0 npr3Δ::kanMX4 pRS416::GPDp-Htt103Q-mCherry(URA)*	npr3Δ		This study
yAF591	tco89Δ + Htt103Q-mCherry	*MATa his3Δ1 leu2Δ0 ura3Δ0 met15Δ0 tco89Δ::kanMX4 pRS416::GPDp-Htt103Q-mCherry(URA)*	tco89Δ		This study
yAF592	his3Δ + Htt103Q-mCherry	*MATa his3Δ1 leu2Δ0 ura3Δ0 met15Δ0 his3Δ::kanMX4 pRS416::GPDp-Htt103Q-mCherry(URA)*	his3Δ		This study
yAF593	npr3Δ + pRS416	*MATa his3Δ1 leu2Δ0 ura3Δ0 met15Δ0 npr3Δ::kanMX4 pRS416(URA)*	npr3Δ		This study
yAF594	tco89Δ + pRS416	*MATa his3Δ1 leu2Δ0 ura3Δ0 met15Δ0 tco89Δ::kanMX4 pRS416(URA)*	tco89Δ		This study
yAF595	his3Δ + pRS416	*MATa his3Δ1 leu2Δ0 ura3Δ0 met15Δ0 his3Δ::kanMX4 pRS416(URA)*	his3Δ		This study

Supplementary References

1. Krobitsch, S., andLindquist, S. (2000) Aggregation of huntingtin in yeast varies with the length of the polyglutamine expansion and the expression of chaperone proteins Proc Natl Acad Sci U S A **97**, 1589-1594 10.1073/pnas.97.4.1589
2. Masser, A. E., Kandasamy, G., Kaimal, J. M., andAndréasson, C. (2016) Luciferase NanoLuc as a reporter for gene expression and protein levels in Saccharomyces cerevisiae Yeast **33**, 191-200 https://doi.org/10.1002/yea.3155
3. Schneider, K. L., Nystrom, T., andWidlund, P. O. (2018) Studying Spatial Protein Quality Control, Proteopathies, and Aging Using Different Model Misfolding Proteins in S. cerevisiae Front Mol Neurosci **11**, 249 10.3389/fnmol.2018.00249
4. Fischbach, A., Johns, A., Schneider, K. L., Hao, X., Tessarz, P., andNyström, T. (2023) Artificial Hsp104-mediated systems for re-localizing protein aggregates Nature Communications **14**, 2663 10.1038/s41467-023-37706-3
5. Sikorski, R. S., andHieter, P. (1989) A system of shuttle vectors and yeast host strains designed for efficient manipulation of DNA in Saccharomyces cerevisiae Genetics **122**, 19-27 10.1093/genetics/122.1.19
6. Janke, C., Magiera, M. M., Rathfelder, N., Taxis, C., Reber, S., Maekawa, H. *et al.* (2004) A versatile toolbox for PCR-based tagging of yeast genes: new fluorescent proteins, more markers and promoter substitution cassettes Yeast **21**, 947-962 10.1002/yea.1142
7. Tong, A. H., Evangelista, M., Parsons, A. B., Xu, H., Bader, G. D., Page, N. *et al.* (2001) Systematic genetic analysis with ordered arrays of yeast deletion mutants Science **294**, 2364-2368 10.1126/science.1065810
8. Winzeler, E. A., Shoemaker, D. D., Astromoff, A., Liang, H., Anderson, K., Andre, B. *et al.* (1999) Functional Characterization of the S. cerevisiae Genome by Gene Deletion and Parallel Analysis Science **285**, 901-906 doi:10.1126/science.285.5429.901
9. Huh, W. K., Falvo, J. V., Gerke, L. C., Carroll, A. S., Howson, R. W., Weissman, J. S., andO'Shea, E. K. (2003) Global analysis of protein localization in budding yeast Nature **425**, 686-691 10.1038/nature02026
