# Supplementary material for: mTOR signaling controls protein aggregation during heat stress and cellular aging in a translation- and Hsf1-independent manner
Source: J Biol Chem. 2025 Jan 10;301(2):108172. doi: 10.1016/j.jbc.2025.108172 (PMC11849620; doi:10.1016/j.jbc.2025.108172)
Supplement: Supplementary information 250101 [file mmc4.pdf]

Supplementary information

mTOR signalling controls protein aggregation during heat stress and cellular aging in a translation- and Hsf1-independent manner

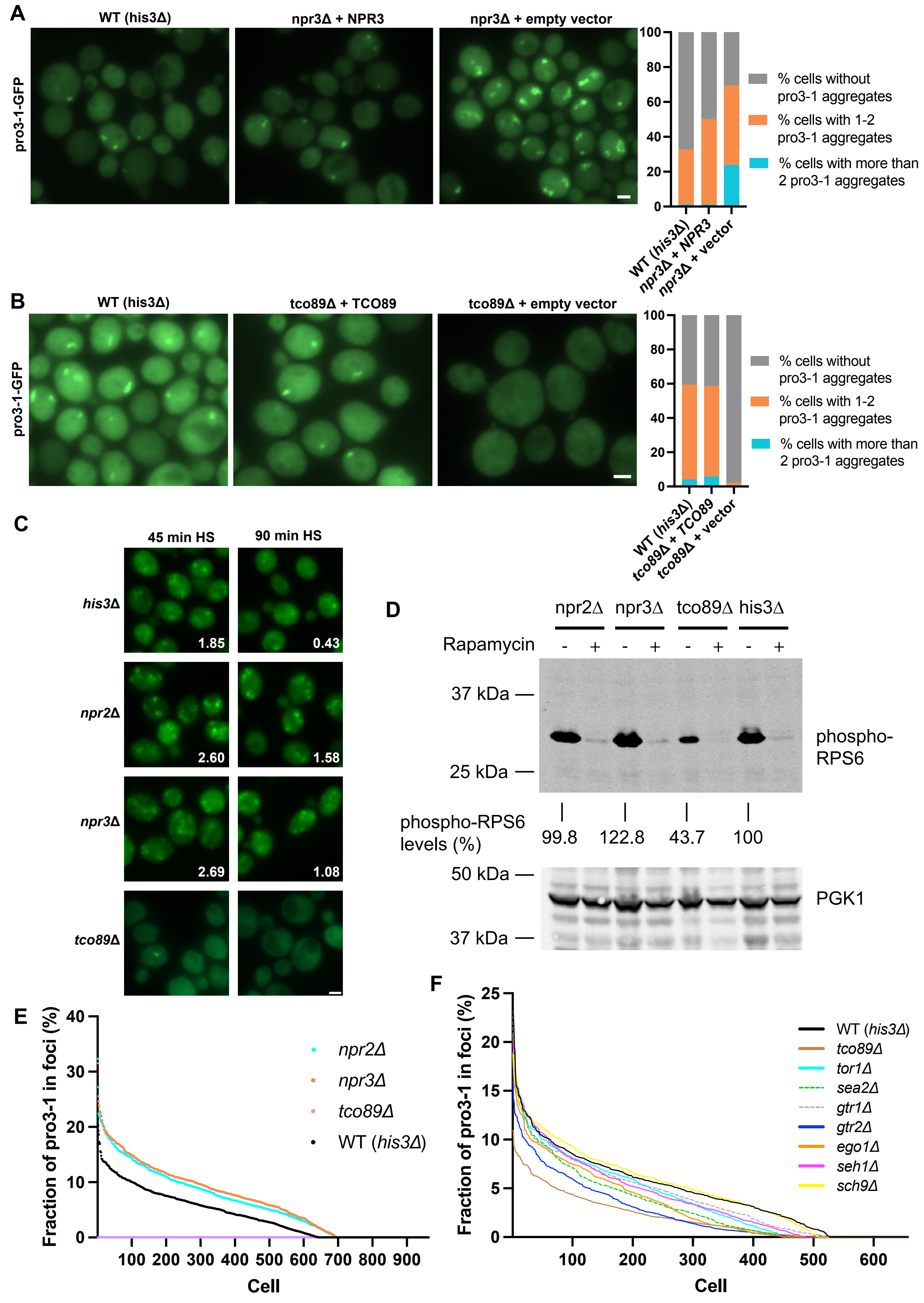
A
WT (his3Δ)
npr3Δ + NPR3
npr3Δ + empty vector
pro3-1-GFP
% cells without pro3-1 aggregates
% cells with 1-2 pro3-1 aggregates
% cells with more than 2 pro3-1 aggregates
WT (his3Δ)
npr3Δ + NPR3
npr3Δ + vector
B
WT (his3Δ)
tco89Δ + TCO89
tco89Δ + empty vector
pro3-1-GFP
% cells without pro3-1 aggregates
% cells with 1-2 pro3-1 aggregates
% cells with more than 2 pro3-1 aggregates
WT (his3Δ)
tco89Δ + TCO89
tco89Δ + vector
C
45 min HS
90 min HS
his3Δ
1.85
0.43
npr2Δ
2.60
1.58
npr3Δ
2.69
1.08
tco89Δ
D
npr2Δ npr3Δ tco89Δ his3Δ
Rapamycin - + - + - + - +
37 kDa
25 kDa
phospho-RPS6
phospho-RPS6 levels (%)
99.8 122.8 43.7 100
50 kDa
37 kDa
PGK1
E
Fraction of pro3-1 in foci (%)
Cell
npr2Δ
npr3Δ
tco89Δ
WT (his3Δ)
F
Fraction of pro3-1 in foci (%)
Cell
WT (his3Δ)
tco89Δ
tor1Δ
sea2Δ
gtr1Δ
gtr2Δ
ego1Δ
seh1Δ
sch9Δ

Figure S1 | Identifying modulators of pro3-1 protein aggregation. A, Complementation analysis of *npr3Δ* cells with a genomically integrated *NPR3* gene. Left: Representative fluorescence microscopy images. Right: Quantification of the percentage of cells with pro3-1 aggregates. *n* = 1 experiment**. B,** Complementation analysis of *tco89*Δ cells with a genomically integrated *TCO89* gene. Left: Representative fluorescence microscopy images. Right: Quantification of the percentage of cells with pro3-1 aggregates. *n* = 1 experiment**. C,** Representative fluorescence microscopy images of cells expressing pro3-1. Cells were subjected to 45 or 90 min continuous 38 °C HS. In the bottom right of the images the amount of counted pro3-1 aggregates are displayed taken from the screen data output (Supplementary Data 1 and 2). Aggregate counting data for *tco89*Δ cells can be found for 90 min HS in Fig. 1D and for 45 min HS in Figs. 2E-F. **D,** phospho-RPS6 levels of *npr2*Δ, *npr3*Δ, *tco89*Δ and control (*his3*Δ) cells as determined by Western Blot. Cells were treated with 219 nM Rapamycin where indicated. Yeast cells in log-phase were collected, lyzed and subjected to SDS-PAGE. **E,** Single-cell analysis of the fraction of pro3-1 in foci after a 90 min continuous 38 °C heat shock in yeast cells with gene deletions in the mTOR signaling regulators NPR2, NPR3 or TCO89 expressing pro3-1-GFP. For this calculation, the pro3-1 intensity sum of the pixels in the aggregates of one cell were divided by the pro3-1 intensity sum of all pixels in the respective cell. For percentage values a multiplication by 100 was performed. *n* = 3 independent experiments with 200-400 cells per strain and per experiment. All cells from the three independent experiments were pooled. In total, exactly 964 cells per strain were analyzed and shown. **F,** Single-cell analysis of the fraction of pro3-1 in foci after a 45 min continuous 38 °C heat shock in deletion strains of more positive mTOR regulators. The same calculation procedure as in E was used. *n* = 3 independent experiments with 100-300 cells per strain and per experiment. All cells from the three independent experiments were pooled. In total, exactly 657 cells per strain were analyzed and shown. Scale bars represent 2 µm.

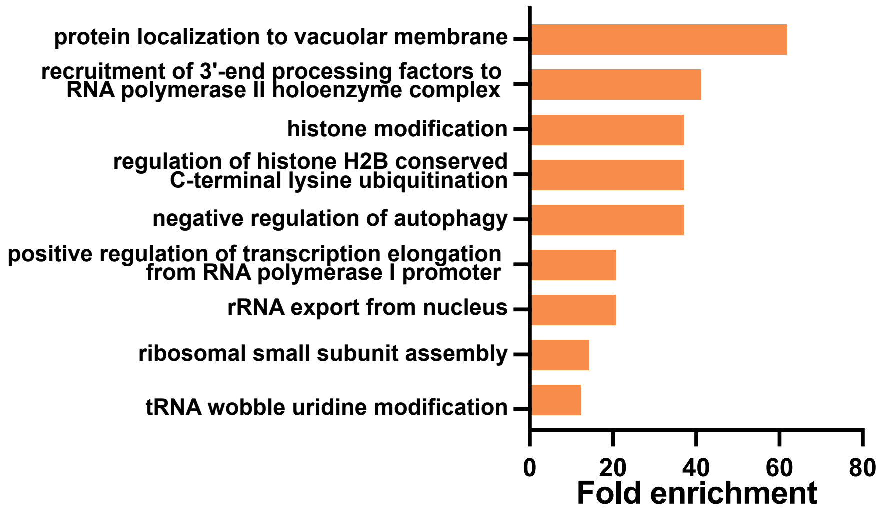

Figure S2 | GO analysis of biological process enrichment with the hits from the screen for identifying genes promoting pro3-1 aggregation. Significant terms with at least 10-fold enrichment are displayed.

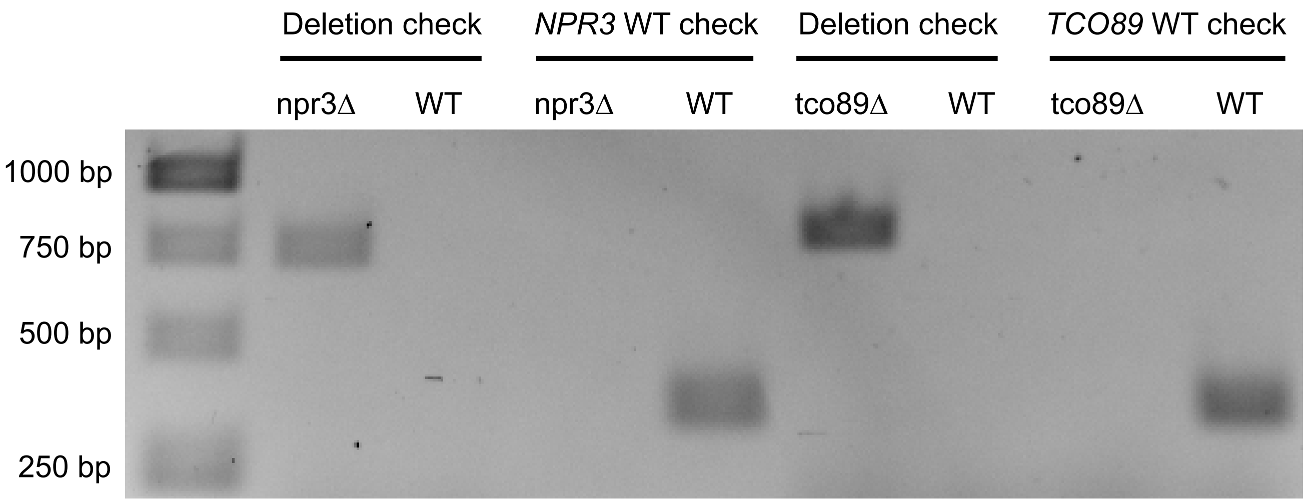

Figure S3 | Agarose gel electrophoresis image. Colony PCR was performed using oligonucleotides specific for the KanMX deletion cassette and for the 5' UTR of *NPR3* or *TCO89* to validate the presence of the deletion cassette (Deletion check). To validate the absence of the WT gene, oligonucleotides specific for the WT ORF sequence of *NPR3* (*NPR3* WT check) or *TCO89* (*TCO89* WT check) to were used. (Used oligonucleotides: KanMX check R: CTGCAGCGAGGAGCCGTAAT; NPR3 5'UTR CHK F: TCCAAAAGAAAGCTGAAATG; TCO89 5'UTR CHK F: GTCTGATCGAGAGGAATTTG; NPR3 WT CHK F: GCCAATCACCTCATCATTAC; NPR3 WT CHK R: TTTTGTGCTTGGATTTTCTC; TCO89 WT CHK F: GTCGAGAGCAAAGAGTAACG; TCO89 WT CHK R: TTTAGATTCTTGCGCATCTC).

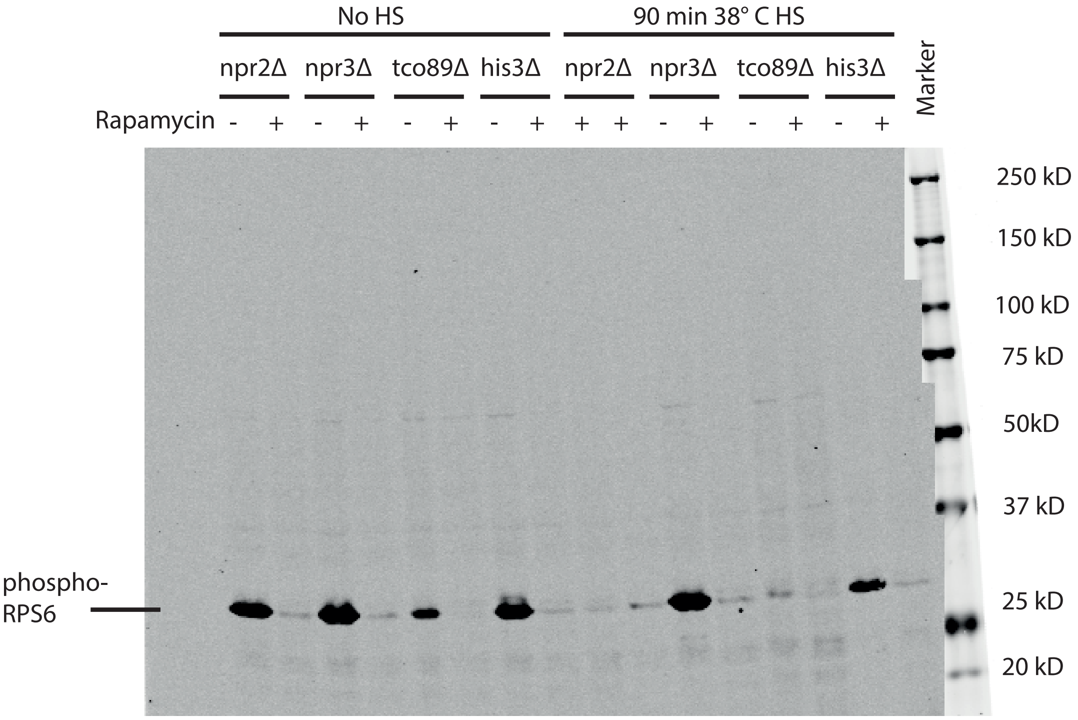

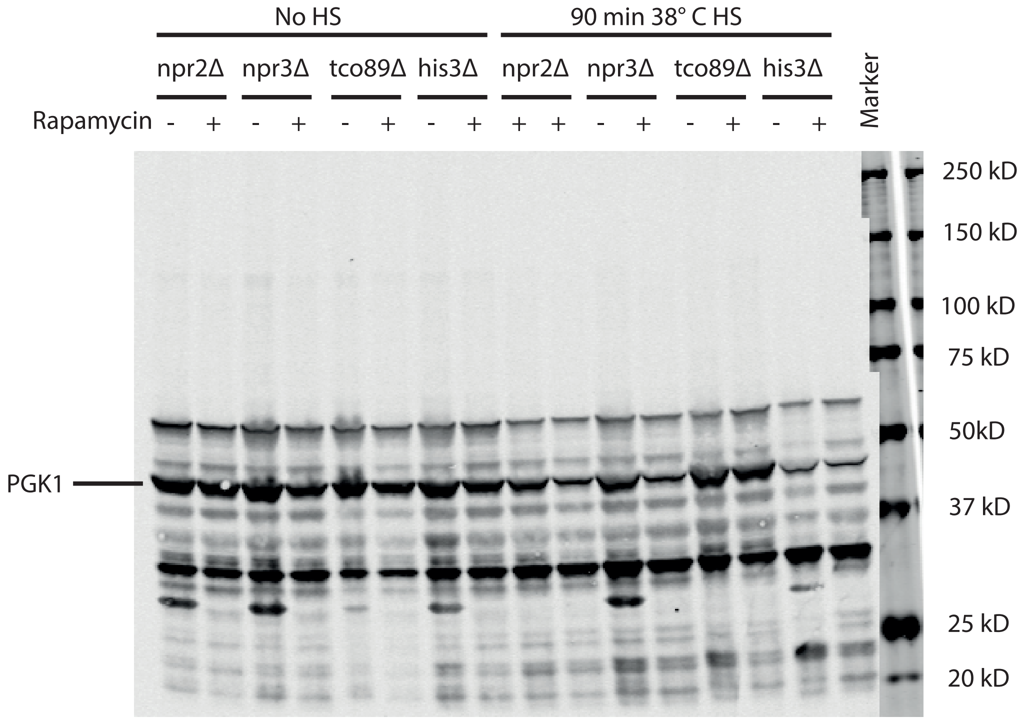

Figure S4 | Uncropped Western Blots from Figure S1D.

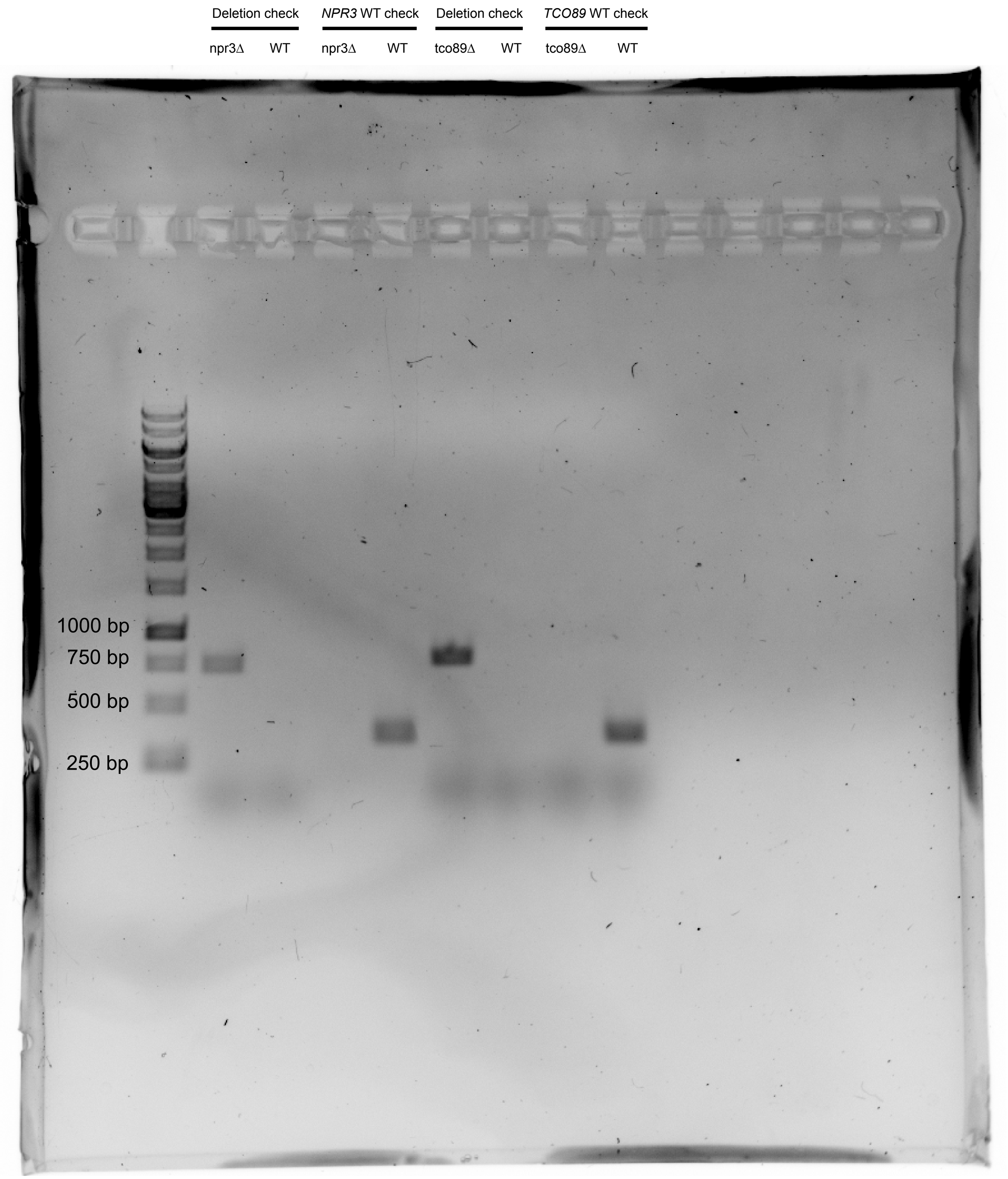

Figure S5 | Uncropped Agarose gel electrophoresis from Figure S3.
